# Cross-Modal Matching of Audio-Visual German and French Fluent Speech in Infancy

**DOI:** 10.1371/journal.pone.0089275

**Published:** 2014-02-20

**Authors:** Claudia Kubicek, Anne Hillairet de Boisferon, Eve Dupierrix, Olivier Pascalis, Hélène Lœvenbruck, Judit Gervain, Gudrun Schwarzer

**Affiliations:** 1 Department of Developmental Psychology, Justus-Liebig-University Giessen, Giessen, Germany; 2 Laboratoire de Psychologie et Neurocognition, CNRS UMR 5105, Université Pierre Mendès France, Grenoble, France; 3 Speech and Cognition Department - GIPSA-lab, CNRS UMR 5216, Université de Grenoble, Grenoble, France; 4 Laboratoire Psychologie de la Perception, Université Paris Descartes, Sorbonne Paris Cité, Paris, France; 5 Laboratoire Psychologie de la Perception, CNRS UMR 8158, Paris, France; Goldsmiths, University of London, United Kingdom

## Abstract

The present study examined when and how the ability to cross-modally match audio-visual fluent speech develops in 4.5-, 6- and 12-month-old German-learning infants. In Experiment 1, 4.5- and 6-month-old infants’ audio-visual matching ability of native (German) and non-native (French) fluent speech was assessed by presenting auditory and visual speech information sequentially, that is, in the absence of temporal synchrony cues. The results showed that 4.5-month-old infants were capable of matching native as well as non-native audio and visual speech stimuli, whereas 6-month-olds perceived the audio-visual correspondence of native language stimuli only. This suggests that intersensory matching narrows for fluent speech between 4.5 and 6 months of age. In Experiment 2, auditory and visual speech information was presented simultaneously, therefore, providing temporal synchrony cues. Here, 6-month-olds were found to match native as well as non-native speech indicating facilitation of temporal synchrony cues on the intersensory perception of non-native fluent speech. Intriguingly, despite the fact that audio and visual stimuli cohered temporally, 12-month-olds matched the non-native language only. Results were discussed with regard to multisensory perceptual narrowing during the first year of life.

## Introduction

From birth on, infants experience a multisensory world where they are required to process information presented in more than one sensory modality, for example, the auditory and visual speech information emanating from the face of a speaker. The multimodality of speech is typically evidenced by the McGurk effect in which conflicting auditory and visual speech information of syllables lead to illusory percepts in adults and children indicating audio-visual speech integration [1]. Remarkably, McGurk-type effects have even been found in 4.5-month-old infants [2], [3], [4]. However, it is still not fully understood when and how infants master the task of matching speech information from different modalities. When visual and auditory speech information is presented simultaneously in an intermodal modal matching task, it has been observed that from 2 months of age infants audio-visually match *vowels* [5], [6], [7], [8], [9], [10]. Furthermore, when visual and auditory stimuli are presented sequentially, that is, across a temporal delay, 6-month-olds were shown to match isolated auditory and visual attributes of *syllables* indicating that temporal synchrony is not essential for matching audio and visual speech information in infants at that age [11]. Moreover, the authors provided evidence for intersensory perceptual narrowing in 11-month-olds, who showed audio-visual matching for their native language syllables only. Despite the fact that in everyday life infants are confronted with *fluent speech* rather than single *vowels* or *syllables*, there is currently little research on the intersensory perception of native and non-native *fluent speech*. One of the few studies addressing this issue suggests that the intersensory response to audio-visual fluent speech emerges late in infancy restricted to native language input [12].

In the present study, we aimed at further studying when and how infants’ ability to perceive the intersensory relation of audible and visible fluent speech develops within the first year of life. In particular, we examined to what extent the *absence* and *presence* of *temporal synchrony* plays a role in infants’ ability to detect the intersensory relation, in both fluent native and non-native audio-visual speech stimuli. An additional goal was to ascertain *whether* and *when* intersensory perceptual narrowing occurs. Therefore, the present study investigated 4.5-, 6- and 12-month-old German-learning infants’ ability to audio-visually match German and French fluent speech.

Because infants are exposed to talking faces on a daily basis, it seems plausible that intermodal representations of face and voice exist early in life [13], [14]. Indeed, recent findings suggest the presence of an early system that detects synchrony and may facilitate the matching of seen and heard speech [8], [15], [16], [17], [18], [19]. With respect to *short speech segments*, there is robust evidence that infants aged 4.5 to 5 months match equivalent information in simultaneously seen and heard vowels [5], [6], [7]. These studies used an intermodal matching task [20], whereby infants were presented with two side-by-side video images of a woman silently articulating the vowels/i/and/a/while the corresponding sound of one vowel was simultaneously played through a centrally placed speaker. It was found that infants looked longer at the face articulating the vowel that matched the sound, which indicates that infants perceived the intersensory coherence of vowel’s audible and visible speech information. These results were even replicated with different vowels [9], with a non-native vowel [9], and were also found in 5- to 6-month-olds for specific disyllables [21].

Because infants find themselves in a socially-rich environment where they are exposed to face-to-face communication from birth on, they experience native audio-visual speech in the form of fluent sequences of utterances. For faces uttering fluent speech, it has been demonstrated that infants at 2.5 to 5 months prefer audio-visually synchronized speech over speech that is out-of-synchrony [17], indicating that infants detect asynchrony between lip movements and speech. Sensitivity to the face-voice relationship for gender emerges between 4 to 6 months of age [22]. Five- to 7-month-olds were found to match fluent speech to faces with one of two affective expressions [23]. Likewise, Pickens et al. [24] found that 3- and 7-month-olds, but not 5-month-olds, perceived the intersensory relation of audible and visible fluent speech, when infants were exposed to two *different* side-by-side faces uttering different stories in the same (native) language along with the audio of one corresponding face.

One of the few studies examining infants’ ability to audio-visually match fluent speech of *different* languages had been conducted by Dodd and Burnham [25], who presented English-learning infants with a *live presentation* of two side-by-side faces belonging to different women, one miming a Greek passage and the other a semantically equivalent English passage with either the appropriate Greek or English audio played simultaneously. Infants at 5 months of age only matched English, their native language, with the corresponding face. This study probably indicates the salience of infants’ native speech to their matching ability. However, different faces were used providing the infants with additional vocal and facial identity cues. It is therefore interesting to extend this study by using one bilingual speaker’s face presented side-by-side.

To resume, infants aged 2 to 6 months have been found to perceive the audio-visual coherence of *short speech segments*. With respect to *fluent speech*, infants as young as 3 months seem to be sensitive to the face-voice synchrony of *native* audio-visual speech.

However, the intermodal matching tasks used in the aforementioned studies provided the infants with auditory and visual information at the same time. Under these conditions, redundant intersensory amodal information (e.g., tempo, intensity) can become highly salient to infants and can enhance their attention to stimuli [26], [27]. Selective attention toward redundant events might then facilitate intersensory matching. To determine whether infants can match auditory and visual speech by extracting intersensory relations at a higher level (e.g., phonetic information), sequential rather than simultaneous presentation of stimuli is necessary. Sequential stimulus presentation rules out the possibility that infants may detect sound-face matching based on audio-visual synchrony, that is, purely temporal grounds.

Pons et al. [11] applied such a variant of the intersensory matching procedure and examined infants’ cross-modal matching of visually and auditorily presented syllables. They compared 6- and 11-month-old English- and Spanish-learning infants’ preferential looking to side-by-side silent videos of a bilingual Spanish-English woman pronouncing the syllables “ba” on one side and “va” on the other side before (2 baseline trials) and after (2 test trials) auditory-only familiarization with either the/ba/or the/va/syllable (2 familiarization trials). Importantly, in this procedure each auditory-only familiarization trial was directly followed by one test trial, respectively. Averaged over the two test trials and compared to looking during baseline trials, looking times of 6-month-old English and Spanish infants were longer at the audio-matching visual syllables, suggesting that they have performed cross-modal matching. But, at 11 months of age, only the English infants did so. As the/ba/vs./va/phonological contrast is known to be perceived by adult English speakers but not by Spanish ones, the fact that older Spanish-learning infants did not match the auditory and visual attributes of non-native speech is interpreted by Pons et al. as suggesting that infants’ sensitivity to intersensory speech narrows down to the native language input during the second half of the first year of life. This conclusion is concordant with the perceptual narrowing/tuning view [28], that is, a tendency for infants to maintain or refine perceptual abilities for native attributes, while declining in discriminating non-native attributes, with which infants have little experience. Such narrowing is well-known and described in many domains, such as cross-species perception of face and voice [29], [30], infants’ face discrimination [31], [32], visual language discrimination [33], and phonetic development [34], [35].

Given that infants match visible and audible syllabic information across a temporal delay, the question arises whether infants also detect the intersensory correspondence for fluent speech in the absence of temporal synchrony cues, that is, when audible and visible speech information is presented sequentially. When does this performance develop in infancy and does it also undergo perceptual narrowing? A recent study by Lewkowicz and Pons [12] addressed these questions by testing groups of 6- to 8-month-old and 10- to 12-month-old English-learning infants with a procedure adapted from Pons et al. [11]. The stimuli consisted of English and Spanish utterances (i.e., they went beyond the syllable level) of one bilingual woman and lasted 30 seconds (visual stimuli) and 20 seconds (audio stimuli). The authors report that none of the age groups showed a visual preference for either language during the baseline condition. During the test trials, only the 10- to 12-month-olds group looked longer at the non-native (Spanish) visual speech after they were familiarized with auditory speech in their native language (English). The fact that 10- to 12-month-old infants did not show a preference for the audio-matching language, but rather for Spanish after listening to English, was interpreted as a novelty preference restricted to auditory native language input due to perceptual narrowing. The 6- to 8-month-olds’ group did not show audio-visual transfer of fluent speech. However, Pons et al. [11] showed in a similar cross-modal task that 6-month-olds matched audio-visual syllables. The question therefore arises whether the processing of fluent speech in the absence of synchrony is too demanding for infants at this age. Nonetheless, it is unclear whether the infants indeed were not capable of matching audible and visible fluent speech. They might have been able to perform the matching but their ability might have been hidden. Especially, methodological issues need to be considered such as, for example, relatively short familiarization times (20 seconds per familiarization trial), and the testing of a broad age group comprising 6- to 8-month-olds, who could have responded to the stimuli in a different manner. In Weikum et al.’s [33] study, for example, it has been demonstrated that 8-month-olds were not able to discriminate between different languages presented visually-only. Thus, it could be speculated that the 8-month-olds of the 6- to 8-month-olds’ sample could have biased the results. Indeed, Weikum et al. [33] demonstrated that 4- and 6-month-old infants are able to extract sufficient visual information from visually-only fluent speech to discriminate between two languages. This leads to the hypothesis that 4- and 6-month-old infants might be able to achieve the matching task, because they may be attentive to the relevant matching cues. However, this assumption is complicated by the fact that in contrast to the 6- to 8-month-old group of Lewkowicz and Pons’ study [12], 10- to 12-month-olds were shown to be responsive to audio-visual fluent speech. A speculation could be that different underlying mechanisms (e.g., qualitatively different processing of matching cues) at different developmental stages might mediate the matching performance during infancy [36], [37], [38], [39]. In fact, development consists of a variety of dynamic processes comprising continual representational changes [40], that may result in u-shaped functions [41]. It is therefore plausible to assume that the processing of audio-visual fluent speech might not always entail monotonic increases across age.

### Aims

The first objective of the present study was to determine when and how the ability to cross-modally match *fluent speech* develops in infancy. Specifically, we aimed at examining whether young infants at 4.5 and 6 months of age exhibit matching of audio and visual fluent speech stimuli in the absence of temporal synchrony cues. Therefore, in a first experiment, we tested 4.5- and 6- month-old German-learning infants’ ability to match heard and seen German and French fluent speech when audio and visual stimuli were presented sequentially. A second experiment intended to investigate the role of temporal synchrony cues regarding the matching performance of heard and seen German and French fluent speech in 6-month-old German-learning infants. Additionally, an older age group comprising 12-month-olds were tested in order to uncover possible developmental changes in the response to audio-visual fluent speech.

## Experiment 1a

In Experiment 1a, we investigated the development of the ability to perform cross-modal matching of audio and visual German and French fluent speech stimuli in infancy. To address this issue, we used a variant of the intersensory matching procedure [11], [12] and compared 4.5- and 6-month-old German-learning infants’ preferential looking to faces silently uttering fluent speech, in German (native) and French (non-native), before (baseline trials) and after (test trials) auditory-only familiarization trials with one of the two languages, respectively. Based on the assumption that infants’ looking behavior indicates cross-modal matching, infants were considered to audio-visually match fluent speech if they exhibited longer looking times to the audio-matching visual language during the test trials as compared to baseline. We predicted that infants of both age groups would match native as well as non-native speech.

### Method

#### Ethics statement

The present study was conducted in accordance to the German Psychological Society (DGPs) Research Ethics Guidelines. The Office of Research Ethics at the University of Giessen approved the experimental procedure and the informed consent protocol. Written informed consents were obtained from the infants’ parents prior to their participation in the study.

#### Participants

The sample consisted of a total of 96 monolingual German-learning infants. All infants were full-term with no visual or auditory deficits, as reported by parents. The data from 7 additional infants were discarded from the final sample due to equipment failure (*n* = 2) or due to extreme fussiness (*n* = 5). The participants were assigned to two age groups: 4.5-month-olds (*n* = 48; mean age = 137.8 days; *SD* = 7.7 days; 26 females), and 6-month-olds (*n* = 48; mean age = 195.6 days; *SD* = 9.4 days; 23 females).

#### Stimuli

The same stimuli were used as in Kubicek et al. [42]. Visual stimuli were silent video clips of four female bilingual German-French speakers. Recording took place in France (Grenoble) for two speakers and Germany (Giessen) for the other two. The speakers were recorded against a blue background, looking directly into a camera with a neutral expression, and reciting French and German sentences adapted from the nursery rhyme “Goldilocks and the three bears”. All videos were matched in image size and time duration. Each of the 30-second video clips showed a full-face image of the speaker and measured 20.6 cm x 18 cm when displayed side-by-side on the monitor, separated by an 11-cm gap. Both videos, French and German, were edited to make sure that they started on a closed mouth and the first mouth opening was synchronized. Audio stimuli were the 30-seconds soundtracks extracted from video recordings, resulting in four different voices speaking either French or German. Sound was presented at conversational sound pressure level (65 dB ±5 dB).

#### Procedure and apparatus

Each infant was tested individually in a baby lab, the caregiver sitting on a chair with the infant on his/her lap. To prevent parents from influencing the looking behavior of their infants, they were told to keep their eyes closed and to refrain from talking for the duration of the experiment. The infants were seated on the caregiver’s lap at a distance of 60 cm in front of a 22-inch monitor (resolution: 1280×1024 pixels). Stimuli were presented by using E-Prime 2.0 software (Psychology Software Tools, Sharpsburg, PA).

Importantly, in this procedure the sound was not presented at the same time as the visual stimuli to ensure that audio-visual synchrony was not mediating intersensory matching.

**Figure 1:**
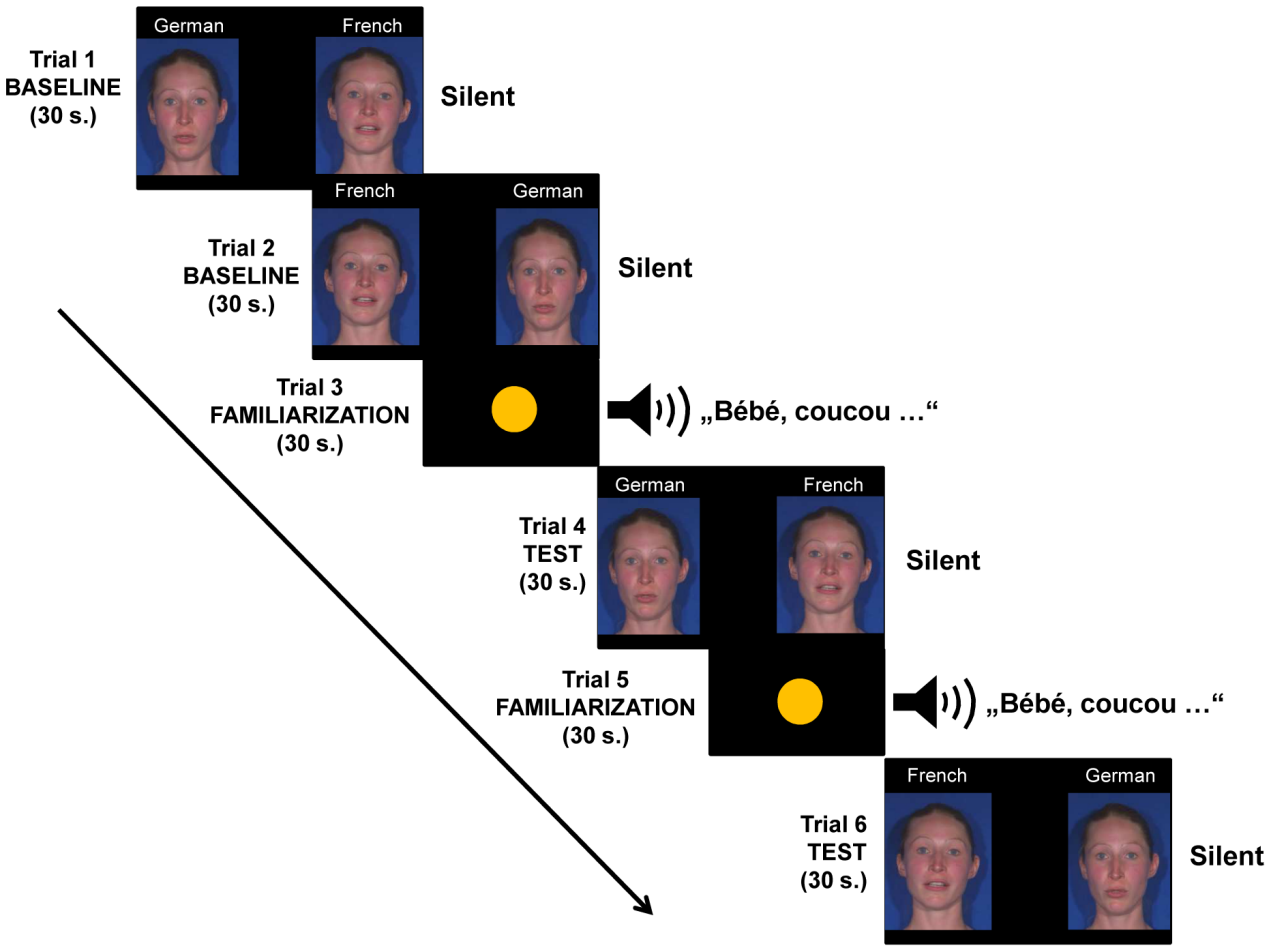
Schematic representation of the procedure used in Experiment 1a. Only the French auditory condition is shown. The model has given written informed consent, as outlined in the PLOS consent form, to publication of their photograph.

There were six 30-second trials (see Figure 1): the first and second trials (baseline condition), infants were presented with two side-by-side silent video clips, displaying one bilingual speaker uttering the same story in French on one side and in German on the other side. The left-right position of French and German videos was counterbalanced across infants in the first trial and reversed in the second one. In the third trial (auditory familiarization trial), infants were presented with the sound stimuli while they were watching an attention getter. Infants were randomly assigned to one of two auditory condition groups, that is, German or French. In the 4^th^trial (test trial), we presented the two initial silent videos again. The 5^th^ and 6^th^ trials were a repetition of the auditory familiarization and test trial, respectively, but the left-right presentation of the silent videos was reversed in the 6^th^ trial. This split test procedure was used because auditory and visual speech information was presented one after the other. To counterbalance the test videos for side two test trials were presented [11], [12]. Based on the expectation that infants would directly match previously heard speech to the corresponding visible facial gestures, each test trial immediately followed each auditory-only familiarization trial.

In sum, the above described procedure first started with a silent baseline condition (including two 30-second trials) that lasted 60 seconds in total, followed by the familiarization-test condition, which was repeated once and had a duration of two minutes in total, containing two 30-second familiarization trials and two 30-second silent test trials, which lasted 60 seconds in total, respectively.

The voices and silent videos of the four female bilingual German-French speakers were counterbalanced across infants and the specific speaker the infants listened to (in the third and 5^th^ trials) was different from the speaker presented in the silent video clips (seen in the two-first baseline trials and the 4^th^ and 6^th^ trials). This ensured, like in Lewkowicz and Pons [12] that any cross-modal preference found was not due to an idiosyncratic pronunciation of the speaker in one language. We extended this precaution by showing four faces instead of one [12] to limit the influence of idiosyncratic facial habits or movements that bilingual speakers may have in one language and not in the other.

#### Scoring

A video camera (specialized for low light conditions) was used to film the infants’ eye movements. The film was then digitized and coded frame by frame by two trained research assistants who were naïve to the hypotheses under investigation. One assistant coded the videos of all infants, while a second coder scored 50% of the data to verify the reliability of the codes. Inter-coder reliability exceeded 0.90.

To be considered in the final analysis, during each trial infants had to look at the stimuli for a minimum of 25% of each trial duration and for a minimum of 5% toward each video of the side-by-side stimuli presentation. In all Experiments, all participants met these criteria for inclusion.

We computed four preference scores by dividing the looking time to one face (German talking face or French talking face) by the amount of total looking time (sum of looking times to the German and French talking faces) separately for the baseline and test trials. These scores were then converted to percentages. For all subsequently performed ANOVAs, these four preference scores were then used as two dependent variables, “Baseline” and “Test” depending on the auditory-only familiarization (French, German). These variables only included the *audio-matching* preference scores on either the German or French talking faces in baseline and test trials, respectively.

Because preliminary analyses in all experiments did not reveal any significant effects of infants’ gender or of the bilingual speakers’ identity on infants’ looking times, the data for these two factors were collapsed in all analyses.

### Results and Discussion

To determine whether the infants showed an initial preference for one of the visual speeches, we submitted the mean percentage of looking time toward the French talking face across the baseline trials to one-sample *t-*tests against chance responding (i.e., *t*-test against chance). *T*-tests were performed separately on each age group. The *t*-tests for both the 4.5- and 6-month-old infants revealed an initial preference for French visual speech during the baseline trials (4.5-month-olds: *M = *54.7% for French visual speech, *SD* = 10.4%, *t* [47] = 3.13, *p<*.01; 6-month-olds: *M* = 55.2% for French visual speech, *SD* = 8.9%, *t* [47] = 4.06, *p<*.001).

To determine whether infants showed cross-modal matching, we compared the preference scores of the audio-matching visible language in the test trials to those during baseline. We therefore conducted a mixed ANOVA with “Condition” (baseline, test) as a within-subjects factor, “Auditory Group” (French, German) and “Age” (4.5 months, 6 months) as between-subjects factors. The ANOVA revealed a main effect of Condition, *F*(1, 137) = 6.9, *p*<.01, *µ^2^* = .07, due to higher preference scores in the baseline as compared to test trials. The ANOVA further yielded a significant Age x Condition x Auditory Group interaction, *F*(2, 137) = 3.6, *p*<.05, *µ^2^* = .05, indicating that infants’ ability to cross-modally match heard and seen speech depended on age and on the language they were auditorily familiarized with.

To further analyze the three-way interaction and to determine whether the infants showed a preference for the audio-matching visual speech after auditory familiarization, we submitted the mean percentage of looking time toward the audio-matching talking faces during the test trials to one-sample *t-*tests against chance responding (i.e., *t*-test against chance). Based on our *a priori* prediction of infants’ matching performance, paired two-tailed *t*-tests that compared preferential looking to the audio-matching visible speech during baseline to preferential looking to the audio-matching visible speech during test trials were conducted. *T*-tests were performed separately on each age group and on each auditory condition group (Table 1).

**Table 1:** Mean of Preference scores (%) toward the visual speech (Standard Deviation) across baseline and test trials in Experiment 1a, depending on infants’ age (4.5- or 6-month-olds) and audio language (German or French); auditory-only familiarization lasted 30 seconds.

Age groups	Audio	Visual speech	Baseline Pref.	Test Pref.	paired *t*-test	*t*-test vs. chance
**4.5-month-olds**	***German***	German	44.7 (12.0)	54.6 (7.4)	*p*<.01	*p*<.01
		French	55.3 (12.0)	45.4 (7.4)		
	***French***	German	45.8 (8.8)	40.1 (9.0)		
		French	54.1 (8.8)	59.9 (9.0)	*p*<.05	*p*<.001
**6-month-olds**	***German***	German	44.1 (10.9)	54.9 (8.0)	*p*<.01	*p*<.01
		French	55.9 (10.9)	45.1 (8.0)		
	***French***	German	45.4 (6.5)	45.1 (8.0)		
		French	54.6 (6.5)	54.9 (8.0)	n.s., *p* = .86	*p*<.05

*Note: T*-tests to compare the Preference scores between the audio-matching visual speech across test trials to preferential looking across baseline trials and *t*-tests comparing preference scores across test trials to chance are also represented.

**Figure 2:**
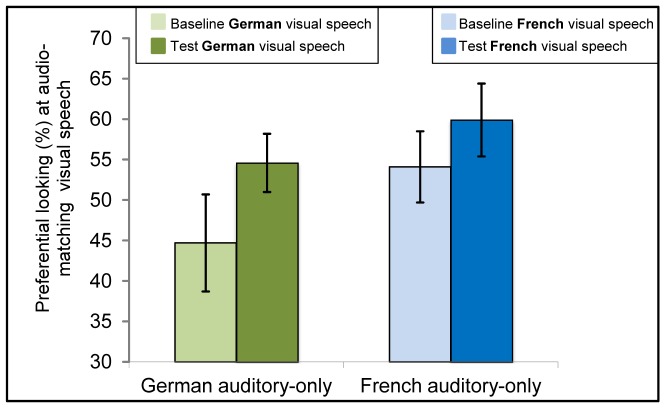
Results of 4.5-month-olds tested in Experiment 1a. Mean of Preference scores at the matching visible speech during baseline and test trials following auditory-only familiarization with either German (green bars on the left, showing preferential looking [%] at the German speaking face during baseline and test trials, respectively) or French (blue bars on the right, showing preferential looking [%] at the French speaking face during baseline and test trials, respectively). Error bars indicate the standard error of the mean.

The *t*-tests revealed cross-modal matching of auditory and visual speech for 4.5-month-old infants’ native, *t*(23) = 3.21, *p*<.01, and non-native language, *t*(23) = 2.3, *p*<.05 (see Figure 2, Table 1).

**Figure 3:**
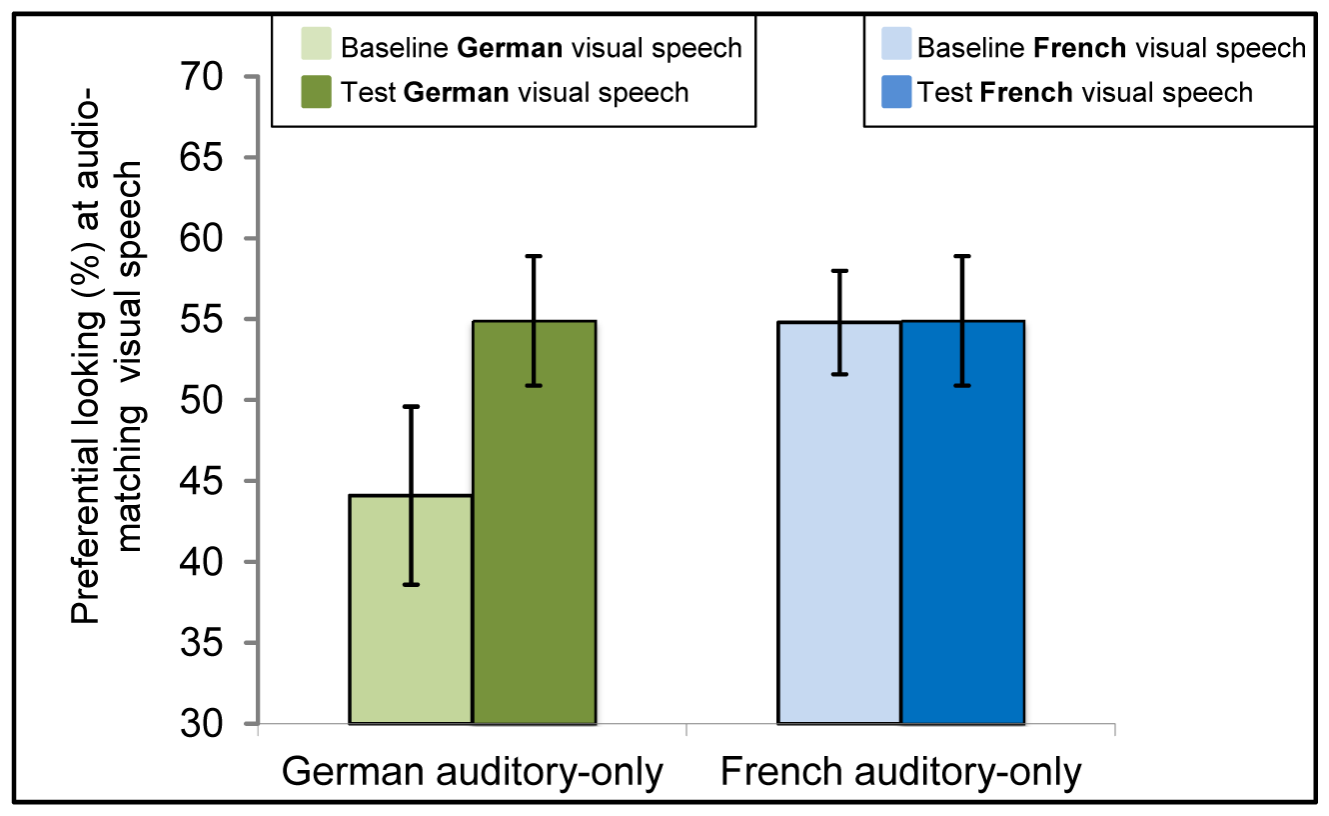
Results of 6-month-olds tested in Experiment 1a. Mean of Preference scores at the matching visible speech during baseline and test trials following auditory-only familiarization with either German (green bars on the left, showing preferential looking [%] at the German speaking face during baseline and test trials, respectively) or French (blue bars on the right, showing preferential looking [%] at the French speaking face during baseline and test trials, respectively). Error bars indicate the standard error of the mean.

Paired two-tailed *t*-tests indicated that 6-month-olds matched their native speech audio-visually, *t*(23) = 3.43, *p*<.01, but not the non-native speech, *t*(23) = 0.17, *n.s.* (see Figure 3, Table 1).

The findings of Experiment 1a demonstrated the ability of 4.5-month-old German-learning infants to cross-modally match audio-visual fluent speech of their native (German) as well as their non-native (French) language. Interestingly, 6-month-old infants have been shown to audio-visually match their native language only. It can be concluded that 4.5- and 6-month-olds recognized and matched auditory and visual speech cues in the absence of temporal synchrony, a remarkable ability.

Moreover, because of the fact that 6-month-olds only showed matching for their native language it could be hypothesized that infants’ ability to detect the correspondence between audible and visible fluent speech narrows down to the native language between 4.5 and 6 months of age. Considered that most of the research demonstrated that infants’ perceptual narrowing in the speech domain occurs later [43] this interpretation should be treated cautiously. However, a potential explanation for this early narrowing may be found in the material we used. The stimuli consisted of lively sentences adapted from a children story and were therefore prosodically-rich. Prosodic cues, including rhythm, intonation, phrasing, are among the cues that infants are able to process at birth (given the availability of prosodic information in-utero [44]). Infants may therefore process prosodic cues earlier than other linguistic cues and may therefore show earlier narrowing for prosodic cues. This could explain why we found earlier narrowing for audio-visual stimuli based on lively passages that contain many prosodic cues.

The finding that 4.5- and 6-month-old infants are able to audio-visually match fluent speech contrasts with the results of Lewkowicz and Pons [12], who did not observe matching of auditory and visual fluent speech in 6- to 8-month-olds. As already mentioned, this might be due to methodological differences, such as testing a broad age group or the duration of familiarization trials. In the study of Lewkowicz and Pons [12], both auditory-only familiarization trials lasted 20 seconds, respectively, whereas the present study used 30 seconds per auditory-only familiarization trial. Experiment 1b aimed to investigate this hypothesis by testing whether 6-month-olds would still be able to demonstrate intersensory matching when they are given less time to become auditory-only familiarized with their native speech.

## Experiment 1b

The purpose of Experiment 1b was to examine whether decreasing the time of auditory-only familiarization from 30 to 20 seconds affects 6-month-olds’ audio-visual matching of fluent native speech.

### Method

#### Participants

The sample consisted of a total of 30 monolingual German-learning 6-month-old infants (*n* = 26; mean age = 198.5 days; *SD* = 7.7 days; 9 females). All infants were full-term with no visual or auditory deficits, as reported by parents. The data from 4 additional infants were discarded from the final sample due to being exposed to another language (*n* = 2) or due to extreme fussiness (*n* = 2).

#### Stimuli and procedure

Stimuli and apparatus were identical to Experiment 1a.

The procedure was identical to Experiment 1a, except that the visual-only trials (baseline and test trials) lasted 30 seconds, whereas both auditory-only trials (familiarization trials) lasted *20* seconds, respectively [12]. We used only the *German (native speech)* auditory condition as Experiment 1a revealed that 6-month-olds failed to match non-native speech.

### Results and Discussion

To determine whether infants performed matching, we conducted a paired two-tailed *t-*test comparing looking at the German speaking face during baseline versus looking at it after auditory-only familiarization (Table 2). No significant finding was obtained, *t*(25) = .03, *n.s.* (*M = *51.1% for German visual speech during test trials, *SD* = 10.4%).

**Table 2:** Mean of Preference scores (%) toward the visual speech (Standard Deviation) across baseline and test trials in Experiment 1b; auditory-only familiarization lasted 20 seconds.

Age group	Audio	Visual speech	Baseline Pref.	Test Pref.	paired *t*-test	*t*-test vs. chance
**6-month-olds**	***German***	German	45.8 (9.2)	48.2 (9.5)	n.s., *p* = .82	n.s., *p* = .79
		French	54.2 (9.2)	51.8 (9.5)		

*Note: T*-tests to compare the Preference scores between the audio-matching visual speech across test trials to preferential looking across baseline trials and *t*-tests comparing preference scores across test trials to chance are also represented.

This indicates that 6-month-old infants did not match their native fluent speech when familiarization times per auditory-only familiarization trial were decreased from 30 to 20 seconds. Thus, the inconsistent findings between Experiment 1a and Lewkowicz and Pons’ study [12] could likely be caused by the use of different familiarization times. It seems as if 6-month-old infants need a sufficient amount of time to encode the auditory language input in order to become able to match it to the visual speech information.

## Experiment 2

Previous studies showed that even newborns are sensitive to temporal synchrony cues, for instance, with respect to short speech segments and non-native vocalizations [15], [19]. Furthermore, infants at 2.5 months of age detect asynchrony between lip movements and speech when watching a talking face [17]. Moreover, studies found that synchrony facilitates the learning of single-syllable and fluent speech in infancy [45], [46], [47].

The second experiment intended to investigate whether temporal synchrony facilitates the ability to match auditory and visual fluent speech in 6-month-old infants. An older group comprising 12-month-olds were additionally tested to ascertain whether 6-month-olds’ matching performance persists into later development.

We used the intersensory matching procedure in which the soundtrack of either the German or French speaking face is presented during the test trials in synchrony with one of the side-by-side videos. We expected 6-month-old infants to benefit from intersensory redundancy, which provides temporal synchrony cues and may thus enhance the salience of audio-visual speech cues. Six-month-old infants should, therefore, match audio-visual fluent speech of German and French language. Infants at 12 months of age were expected to match at least their native, that is, German language due to perceptual narrowing.

### Method

#### Participants

The sample consisted of a total of 88 monolingual German-learning infants. All infants were full-term with no visual or auditory deficits, as reported by parents. The data from nine additional infants were discarded from the final sample due to equipment failure (*n* = 2) or due to extreme fussiness (*n* = 7). There were forty-three participants in the 6-month-old group (mean age = 195.0 days; *SD* = 8.2 days; 20 females), and forty-five participants in the 12-month-old group (mean age = 368.7 days; *SD* = 12.1 days; 28 females).

#### Stimuli

All stimuli were identical to Experiment 1a. Additionally, for audio-visual (i.e., in-sound) stimuli, the same video clips were presented at conversational sound pressure level (65 dB ±5 dB).

#### Apparatus and procedure

After showing an attention getter, we used the intersensory matching procedure.

**Figure 4:**
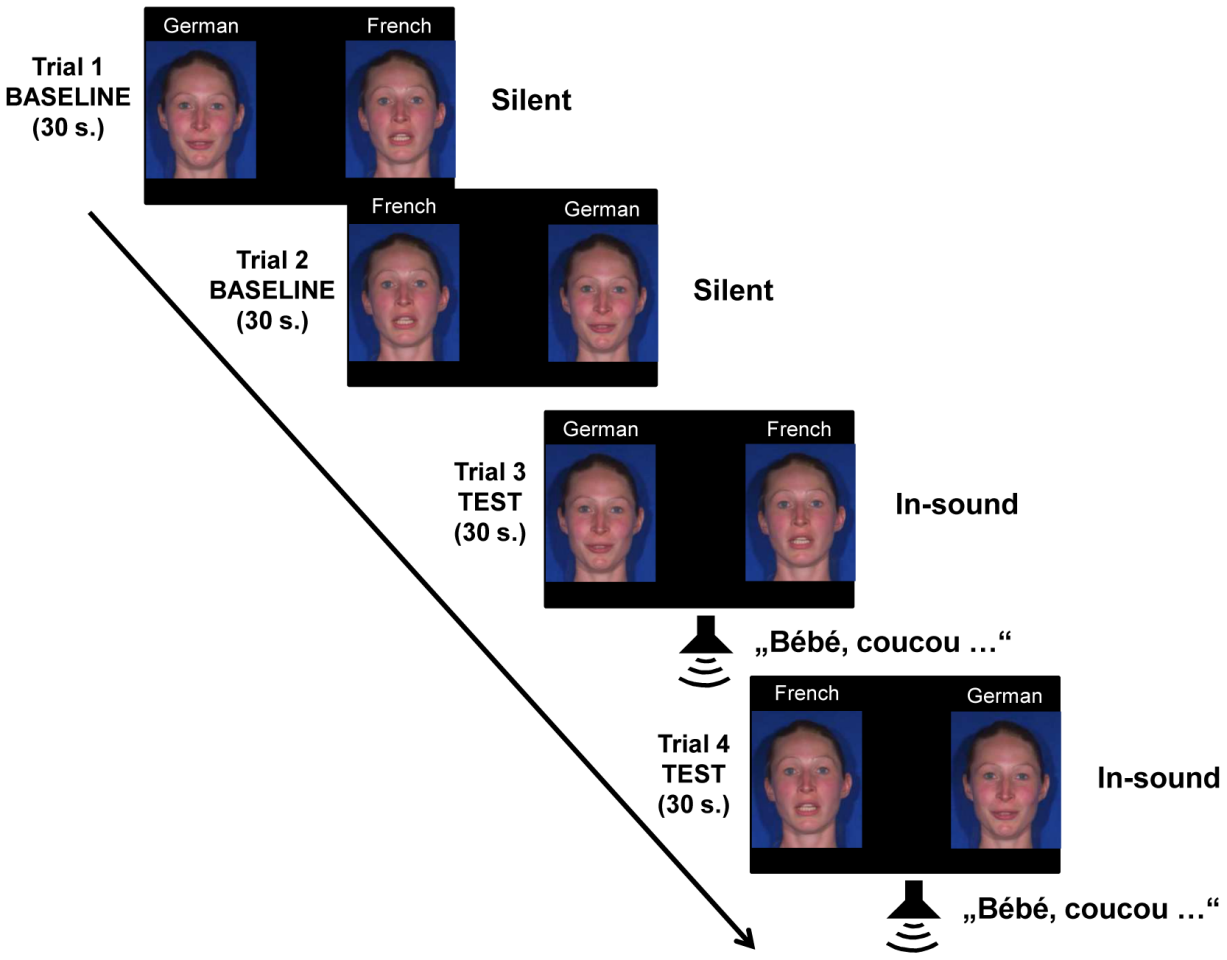
Schematic representation of the procedure used in Experiment 2. Only the French auditory condition is shown. The model has given written informed consent, as outlined in the PLOS consent form, to publication of their photograph.

There were four 30-second trials (see Figure 4): in the first and second trials (baseline condition), infants were presented with two side-by-side silent video clips, displaying one bilingual speaker uttering the same story in French on one side and in German on the other side. The left-right position of French and German videos was counterbalanced across infants in the first trial and reversed in the second one. In the 3^rd^ and 4^th^ trials (test trials), infants were presented with the corresponding voice of either the German or the French speaking face synchronously with the presentation of the silent videos. Infants were randomly assigned to one of the two auditory groups (German or French). The silent and audio-visual videos of the four female bilingual German-French speakers were counterbalanced across infants.

In sum, the above described procedure first started with a silent baseline condition (including two 30-second trials), which lasted 60 seconds in total, followed by the test condition containing two 30-second audio-visual test trials, which lasted 60 seconds in total.

### Results and Discussion

As a first analysis, we determined whether the infants showed an initial preference for one of the visual speeches during the baseline condition. In concordance with the data of the first experiment, the analysis revealed that 6-month-olds showed an inherent preference for French visual speech during baseline trials (*M* = 54.2%, *SD* = 8.4%, *t* [42] = 3.27, *p<*.01; tested against chance), whereas 12-month-olds did not show any preference (*M* = 49.2% for French visual speech, *SD* = 11.1%).

To analyze whether the infants audio-visually matched the languages, we compared the preference scores of the audio-matching visible speech of baseline with the in-sound presented test trials by computing a mixed ANOVA with “Condition” (baseline, test) as within-subjects factor, “Auditory Group” (French, German) and “Age” (6 months, 12 months) as between-subjects factors. The ANOVA found a significant Condition x Age interaction, *F*(1, 84) = 5.02, *p*<.05, *µ^2^* = .06, indicating that 6- and 12-month-old infants showed a differential looking behavior during the baseline trials as already reported above. The ANOVA further yielded a significant Condition x Age x Auditory Group interaction, *F*(1, 84) = 4.5, *p*<.05, *µ^2^* = .05, indicating that infants’ audio-visual matching ability depended on age and on the language they have heard. Similarly to Experiment 1, we submitted the mean percentage of looking time toward the audio-matching talking faces during the test trials to one-sample *t-*tests against chance responding. Based on our *a priori* prediction of infants’ matching performance when temporal synchrony cues were provided, paired two-tailed *t*-tests that compared preferential looking to the audio-matching visible speech during baseline to preferential looking to the audio-matching visible speech during test trials were conducted. *T*-tests were performed separately on each age group and on each auditory condition group (Table 3).

**Table 3:** Mean of Preference scores (%) toward the visual speech (Standard Deviation) across baseline and test trials in Experiment 2, depending on infants’ age (6- or 12-month-olds) and audio language (German or French).

Age groups	Audio	Visual speech	Baseline Pref.	Test Pref.	paired *t*-test	*t*-test vs. chance
**6-month-olds**	***German***	German	45.3 (10.6)	54.6 (13.0)	*p*<.05	*p*<.05
		French	54.7 (10.6)	45.4 (13.0)		
	***French***	German	45.8 (6.0)	38.4 (7.4)		
		French	54.1 (6.0)	61.1 (7.4)	*p*<.001	*p*<.001
**12-month-olds**	***German***	German	51.3 (13.3)	49.4 (8.6)	n.s., *p* = .57	n.s., *p* = .72
		French	48.7 (13.3)	50.6 (8.6)		
	***French***	German	52.3 (7.9)	45.3 (7.5)		
		French	47.7 (7.9)	54.7 (7.5)	*p*<.05	*p*<.01

*Note: T*-tests to compare the Preference scores between the audio-matching visual speech across test trials to preferential looking across baseline trials and *t*-tests comparing preference scores across test trials to chance are also represented.

**Figure 5:**
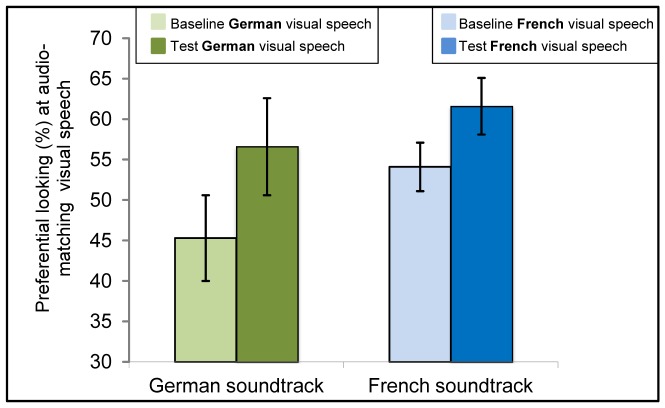
Results of 6-month-olds tested in Experiment 2. Mean of Preference scores at the matching visible speech during baseline and test trials with either German (green bars on the left, showing preferential looking [%] at the German speaking face during baseline and test trials, respectively) or French soundtrack (blue bars on the right, showing preferential looking [%] at the French speaking face during baseline and test trials, respectively). Error bars indicate the standard error of the mean.

The *t*-tests revealed intersensory matching of audio-visual speech for 6-month-old infants’ native, *t*(19) = 2.7, *p*<.05, and non-native language, *t*(22) = 4.1, *p*<.001 (see Figure 5, Table 3).

**Figure 6:**
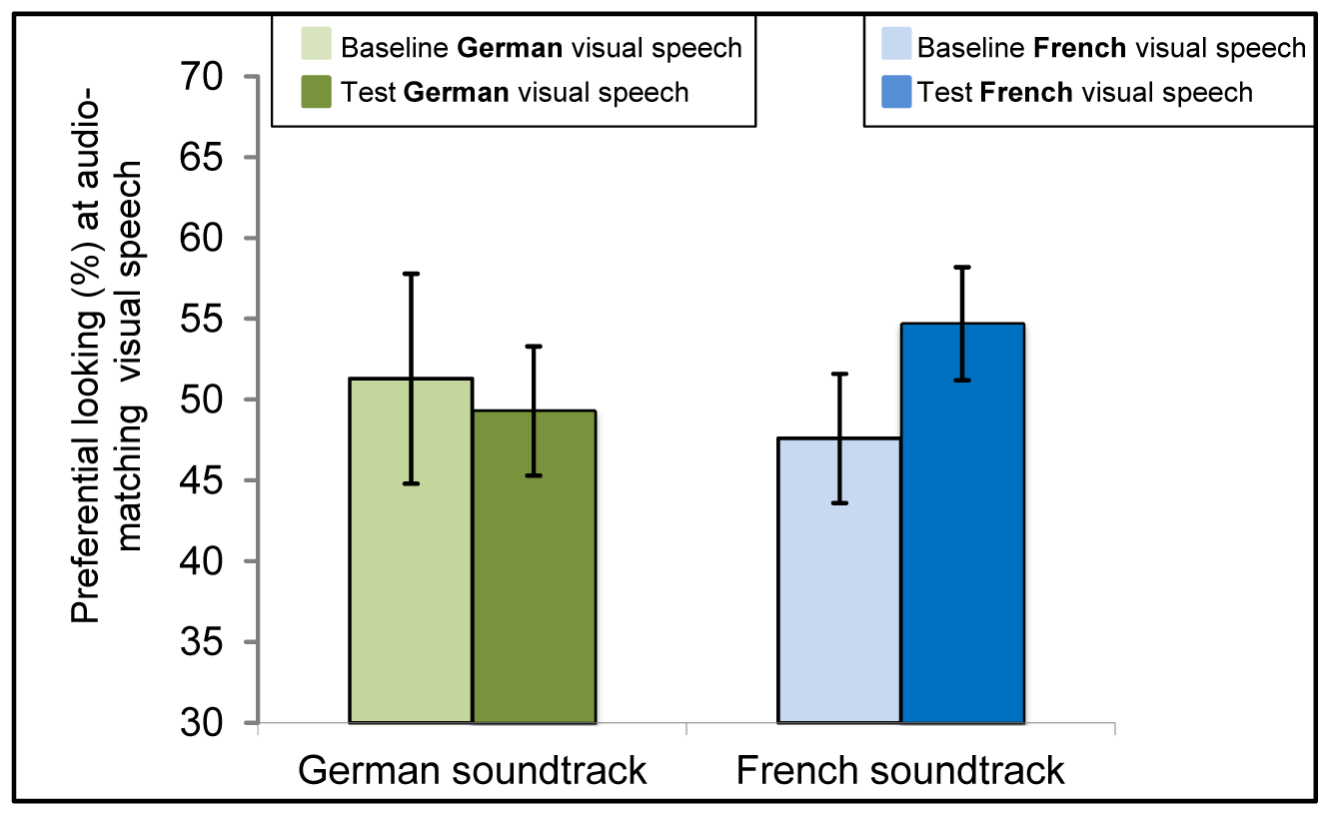
Results of 12-month-olds tested in Experiment 2. Mean of Preference scores at the matching visible speech during baseline and test trials with either German (green bars on the left, showing preferential looking [%] at the German speaking face during baseline and test trials, respectively) or French soundtrack (blue bars on the right, showing preferential looking [%] at the French speaking face during baseline and test trials, respectively). Error bars indicate the standard error of the mean.

Twelve-month-olds were found to look longer at audio-visually presented French videos compared to baseline, *t*(22) = 2.7, *p*<.05. No difference was found in infants audio-visually presented with German videos, *t*(21) = 0.5, *n.s*. Thus, at 12 months of age, infants only matched French, the non-native language (see Figure 6, Table 3).

Results show that given simultaneous audio-visual presentation, German learning 6-month-old infants audio-visually matched native (German) as well as non-native (French) fluent speech. They benefited from the temporal synchrony cues independently of language familiarity. This suggests that even though narrowing might have been at play at this stage, when presented with simultaneous audio-visual stimuli, infants may detect face-sound correspondence by relying on purely temporal information, rather than language-specific prosodic cues. Unexpectedly, 12-month-old infants only matched the non-native language. Although surprising, this finding is in line with eye-tracking studies demonstrating that 12-month-olds attend longer to the mouth region when a face is talking a non-native language [48] or when they previously heard non-native speech [42]. Therefore, we can speculate that the successful matching performance for the non-native speech in 12-month-olds could be explained by differential face-scanning, that is, attending to the mouth area for processing the French stimuli, which in turn helped them to uncover the correspondence between the auditory and visual information.

## General Discussion

The objective of the present study was to investigate when and how the ability to cross-modally match native and non-native fluent speech in the absence and presence of temporal synchrony develops in infants. To investigate these issues, we presented the infants with a baseline (side-by-side silent videos), followed by a familiarization (audio-only) – test (side-by-side silent videos) condition (Experiment 1) or audio-visual test condition (Experiment 2). Based on the assumption that infants’ looking behavior indicates cross-modal matching of audio-visual speech, infants were considered to perform matching if they exhibited longer looking times to the audio-matching visual language during the test condition as compared to baseline.

In the absence of synchrony, 4.5-month-olds audio-visually matched native as well as non-native speech, whereas 6-month-old infants matched their native language only (Experiment 1a). However, this evidence of matching was dependent on the amount of familiarization time provided (Experiment 1b). In the presence of synchrony, 6-month-olds matched native as well as non-native speech. Twelve-month-olds were found to only perceive the coherence of visible and audible speech relations for their non-native language. Overall, these results are consistent with the hypothesis that perceptual narrowing occurs with multisensory fluent speech.

The fact that 4.5- and 6-month-olds showed a visual preference for French stimuli in the baseline condition could be explained by inherent features in the French stimuli. The French stimuli consisted of many more vowels produced with lip protrusion (40) than the German stimuli (16). Protruded lip shapes might be salient and attractive to infants as they resemble lip smacks. Therefore, this excess of rounding might have attracted the younger infants’ attention. The fact that the 12-month-olds in Experiment 2 did not show a visual preference for French stimuli in the baseline could mean that they have already learned to pay attention to several phonetic features including lip protrusion and spreading and are not as much attracted by rounded lip shapes.

The intersensory matching of native and non-native fluent speech in 4.5-month-old infants is consistent with studies demonstrating that they audio-visually match short speech segments [5]. Our results are also in line with the finding that infants at 4.5 months of age exhibit the McGurk effect [2], [3], [4]. Our findings, therefore, extend previous research and provide evidence of the ability to match audible and visible information of native and non-native fluent speech in the absence of synchrony in infants as young as 4.5 months of age. This is remarkably earlier than would have been expected and suggests that despite their poor linguistic knowledge, 4.5-month-olds seem to be able to process some auditory and visual speech cues that sufficiently help them to master the matching task. Future research is needed to identify the relevant matching cues, but common amodal relations (e.g., tempo, duration, and intensity) of audible and visible speech (facial movements) are likely to be implicated [49].

Our findings of the 6-month-olds are congruent with prior research demonstrating matching of isolated audio-visual syllables without temporal synchrony cues in infants at 6 months of age [11], but differ from Lewkowicz and Pons [12] in that, we demonstrated that 6-month-old infants are able to match audio-visually their native language when given sufficient familiarization, highlighting the importance of the familiarization time in this kind of paradigm. Yet, it is still unclear whether the results of Lewkowicz and Pons’ 6- to- 8-month-olds who did not to show matching was partly caused by including 8-month-olds into the sample as the current study emphasizes that the duration of familiarization has an impact on 6-month-olds’ matching performance.

Why are 6-month-olds not matching French? One explanation could be that 6-month-old infants might only have extracted specific prosodic and phonetic auditory and visible cues based on their daily experience with the native language. It could be hypothesized that infants may have already undergone some multisensory perceptual narrowing by this age. This interpretation, however, does not fit easily into current research devoted to perceptual narrowing suggesting that infants’ narrowing may be complete by the end of the first year, which is later as proposed in the current study. However, with respect to the speech domain, few studies indicate an earlier timing [43]. For instance, there is evidence that the decline of discriminating non-native vowels might begin earlier, between 6 and 8 months of age [50], [51]. Moreover, Lewkowicz and Ghazanfar [29] found intersensory narrowing for non-native vocalizations between 6 and 8 months of age. Because of the fact that only unisensory response to vowels, or intersensory response to other-species vocalisations was studied, the results of these two studies are difficult to compare to the topic of the current study, which focused on human audio-visual fluent speech. However, our use of richer linguistic material might in fact have pushed the narrowing ability. Because of the availability of prosodic information in utero, newborns have a long experience of their native-language prosodic patterns. Newborns have even been shown to be able to discriminate speech samples simply based on prosodic cues [44]. Infants may therefore develop prosodic processing abilities earlier than they do for segmental information. They may therefore show earlier narrowing for prosodic cues than segmental cues and may thus show earlier narrowing for audio-visual stimuli based on prosodically-rich passages.

In order to determine the extent to which the interpretation that 6-month-olds’ data do indeed reflect multisensory perceptual narrowing can be generalized, it would be interesting to study French-learning infants’ matching performance of German and French fluent speech. This experiment is currently running in Grenoble (France) and data collection/analysis is not yet finished. Therefore, the results of French infants will be published elsewhere.

If the results of the present study are considered together with the findings of Lewkowicz and Pons [12], it seems that the observed developmental pattern might be inconsistent. Infants as young as 4.5 months were found to match native as well as non-native speech, whereas 6-month-olds perceived the intersensory coherence of their native language only, however, only under the condition that sufficient familiarization time was given. When an age group comprising 6- to 8-month-olds was tested with shorter familiarization, infants did not exhibit a response to intersensory fluent speech, whereas infants toward the end of the first year finally did. These findings could probably point to the hypothesis that an u-shaped function might have driven the underlying developmental processes, as observed, for example, with respect to infants’ face processing [52], [53], phonetic perception [51], and audio-visual perception of native fluent speech [24]. In fact, after auditory-only familiarization, 4.5- and 6-month-olds showed a familiarity preference toward the audio-matching visible speech that significantly differed from looking during baseline. According to studies using this kind of paradigm [11], we interpret this pattern of results as evidence for cross-modal matching. However, the 10- to 12-month-old infants of Lewkowicz and Pons’ study [12] showed a novelty preference for the non-native visible speech after listening to native speech, which indicates that infants had recognized their native language and then moved toward the novel stimulus. Taken together, these differential preferences might reflect the fact that infants in earlier and later developmental stages are difficult to compare regarding their *matching* behavior. At first, they might attend to different features of the stimuli that remain to be figured out. Secondly, speed of information processing and working memory demands that could affect direction of preferences need to be considered.

Indeed, when the soundtrack of the speaking face was presented in synchrony, 6-month-olds finally matched fluent speech for non-native speech. In order to benefit from synchrony between the auditory and visual information of multimodal speech, infants need to orient attention toward the vocal tract where redundant cues are available [54], [55]. At 6 months of age, when entering the canonical babbling stage [56], infants indeed start to attend more to the mouth of speakers [48]; access to complementary audio-visual speech cues might be highly advantageous and may foster imitation in younger infants [10]. Thus, the simultaneous presentation of auditory and visual speech attributes might have facilitated matching performance by providing temporal synchrony cues and by requiring less working memory. Six-month-old infants, therefore, also matched audio-visual fluent speech of their non-native language when given sufficient temporal correspondence information.

Conversely, 12-month-old infants only benefitted from synchrony cues for the non-native speech. Although intriguing, this finding is consistent with current research investigating visual attention to facial regions of audible and silently talking faces [42], [48]. Regarding the processing of audible talking faces, it has been demonstrated that 12-month-old infants showed longer looking times toward the mouth area when a face was talking non-native speech [48]. Moreover, Kubicek et al. [42], by examining the impact of auditory speech on the visual processing of silently talking faces, revealed that after auditory-only exposure to their non-native language, 12-month-olds also looked more at the mouth while looking times at the eyes decreased. We therefore hypothesize that because 12-month-old infants are attuned to their native language [57], [58], their processing of native speech does not necessarily rely on language specific visual cues. On the contrary, when 12-month-olds infants are confronted to non-native, that is, unfamiliar speech, they attend longer to the mouth region, and therefore can benefit from synchrony between auditory and visual speech information and then show matching of auditory and visual speech.

## Conclusions

The current results demonstrated that German learning infants at 4.5 and 6 months of age cross-modally match audio-visual fluent speech when auditory and visible speech information was presented one after the other. The fact that 4.5-month-old infants performed matching independent of language familiarity indicates that, at the *fluent speech level*, synchrony is not essential for matching auditory and visual speech in infants at this age. In contrast, 6-month-olds demonstrated matching for native fluent speech only, which probably suggests that, when using prosodically-rich stimuli, multisensory perceptual narrowing might appear earlier than has been suggested so far.

The finding that 6-month-olds also performed matching for non-native speech when temporal synchrony cues were available can be interpreted in the light of multisensory temporal information processing. Intersensory redundancy might have facilitated infants’ matching as simultaneous audible and visible speech cues become enhanced and highly salient. These complementary or enhanced cues might have driven infants’ matching performance of non-native speech. The findings of the 6-month-olds, who in the absence of synchrony showed evidence of perceptual narrowing, whereas in the presence of synchrony this evidence disappeared, propose the assumption that the presence or absence of intersensory perceptual narrowing might be contingent upon the presence or absence of temporal synchrony.

When simultaneously perceiving visible and auditory speech information 12-month-olds have been found to only match non-native speech. This matching performance might be based on a differential pattern of visual attention toward the mouth region in infants at this age that is dependent on which language is audio-visually spoken to them and, therefore, may reflect multisensory perceptual narrowing [48].

Taken together, the results of the present study further confirm that perceptual narrowing is a domain-general process, which might differ in developmental timing dependent on perceptual input and on task demands.
